# Genetic economy in picornaviruses: Foot-and-mouth disease virus replication exploits alternative precursor cleavage pathways

**DOI:** 10.1371/journal.ppat.1006666

**Published:** 2017-10-02

**Authors:** Morgan R. Herod, Sarah Gold, Lidia Lasecka-Dykes, Caroline Wright, Joseph C. Ward, Thomas C. McLean, Sophie Forrest, Terry Jackson, Tobias J. Tuthill, David J. Rowlands, Nicola J. Stonehouse

**Affiliations:** 1 School of Molecular and Cellular Biology, Faculty of Biological Sciences and Astbury Centre for Structural Molecular Biology, University of Leeds, Leeds, United Kingdom; 2 The Pirbright Institute, Pirbright, Surrey, United Kingdom; University of California, Irvine, UNITED STATES

## Abstract

The RNA genomes of picornaviruses are translated into single polyproteins which are subsequently cleaved into structural and non-structural protein products. For genetic economy, proteins and processing intermediates have evolved to perform distinct functions. The picornavirus precursor protein, P3, is cleaved to produce membrane-associated 3A, primer peptide 3B, protease 3C^pro^ and polymerase 3D^pol^. Uniquely, foot-and-mouth disease virus (FMDV) encodes three similar copies of 3B (3B1-3), thus providing a convenient natural system to explore the role(s) of 3B in the processing cascade. Using a replicon system, we confirmed by genetic deletion or functional inactivation that each copy of 3B appears to function independently to prime FMDV RNA replication. However, we also show that deletion of 3B3 prevents replication and that this could be reversed by introducing mutations at the C-terminus of 3B2 that restored the natural sequence at the 3B3-3C cleavage site. *In vitro* translation studies showed that precursors with 3B3 deleted were rapidly cleaved to produce 3CD but that no polymerase, 3D^pol^, was detected. Complementation assays, using distinguishable replicons bearing different inactivating mutations, showed that replicons with mutations within 3D^pol^ could be recovered by 3D^pol^ derived from “helper” replicons (incorporating inactivation mutations in all three copies of 3B). However, complementation was not observed when the natural 3B-3C cleavage site was altered in the “helper” replicon, again suggesting that a processing abnormality at this position prevented the production of 3D^pol^. When mutations affecting polyprotein processing were introduced into an infectious clone, viable viruses were recovered but these had acquired compensatory mutations in the 3B-3C cleavage site. These mutations were shown to restore the wild-type processing characteristics when analysed in an *in vitro* processing assay. Overall, this study demonstrates a dual functional role of the small primer peptide 3B3, further highlighting how picornaviruses increase genetic economy.

## Author summary

Positive-strand RNA viruses are generally typified by a small genome size. To compensate for this limitation they have evolved ways of multiplying the functions achievable from a small genome, thus increasing ‘genetic economy’. In the picornavirus family of RNA viruses the viral proteins are produced via a series of transient precursors, which are believed to possess functions required for virus replication which are additional to those of the fully processed proteins. In this report, we show that the small 3B3 protein from foot-and-mouth disease virus, an economically important animal pathogen, is key in directing the processing pathways of the P3 polyprotein to facilitate release of the viral polymerase. This is in addition to, and independent from, the role of 3B3 as a primer for replication. This study exemplifies how multiple functions within one small viral protein can control viral replication and demonstrates a new level of genetic economy in the picornaviruses. Furthermore, systems that provide ‘help’ *in trans* to assist replication have the potential to be exploited for the production of attenuated vaccines (e.g. using “helper’ cell lines). We are now in the process of investigating how mutations at polyprotein processing boundaries may be used for producing attenuated vaccines.

## Introduction

The *Picornaviridae* family of single-stranded positive-sense RNA viruses has evolved mechanisms to increase genetic economy and compensate for their relative small genome size. All picornaviral proteins are initially produced as precursors that are progressively cleaved into the final products and some of the processing intermediates have roles in viral replication additional to those of the final proteins [1–16]. Such strategies to maximise genetic economy are not limited to the *Picornaviridae* and are thought to be utilised by a wide range of RNA viruses to expand the functional potential of limited genome sizes (such as reviewed by [17] and [18]).

Members of the *Picornaviridae* include several well-known human pathogens such as poliovirus (PV) and important animal pathogens such as foot-and-mouth disease virus (FMDV). FMDV is the causative agent of foot-and-mouth disease, an economically-significant, acute vesicular disease of cloven-hoofed ruminants that is endemic in many parts of the world [19]. The FMDV genome contains a single open reading frame that is flanked by 5' and 3' untranslated regions (UTRs) and a 3' poly(A) tail [20]. The 5' UTR contains multiple elements essential for viral RNA replication including the internal ribosome entry site (IRES) and a *cis*-acting replicative element (CRE) [21–24]. The open reading frame is translated into a single polyprotein that is proteolytically-processed by viral encoded proteases to generate all the viral structural and non-structural proteins. Primary processing of the polyprotein occurs at three sites to generate four products; L^pro^ [25], the capsid precursor protein P1-2A, and the non-structural protein precursors P2 (2BC) and P3 (3AB123CD) [26]. Many of the details of polyprotein processing are inferred by analogy with PV. The primary products P1-2A, P2 and P3 are subsequently cleaved by 3C^pro^ and/or 3CD, as has been shown for PV [27–34], to produce a series of intermediate precursor products and the final proteins. Processing of the P3 polyprotein occurs via a series of intermediate precursors to generate the viral RNA-dependent RNA polymerase (RdRp), 3D^pol^ [35–37], the viral protease 3C^pro^ [38, 39], three non-identical copies of the 3B (also known as VPg) replication primer (peptides 3B1, 3B2 and 3B3) [40–43], and a transmembrane protein 3A [44, 45]. Of the processing intermediates, 3AB and 3CD are believed to play essential roles in the replication-cycle of PV in addition to those of the final processed proteins [1–14]. FMDV 2BC has been shown to inhibit the secretory pathway [15], with other intermediates probably also involved. In PV, the transient P3 precursor protein has also been implicated as having essential roles in replication [16], and the PV 3CD precursor, which has no polymerase activity, is proteolytically-active but has a different substrate specificity compared to 3C^pro^ [12, 27–29, 46–48].

Picornavirus RNA replication occurs in replication complexes associated with membranous vesicles collectively referred to as “replication organelles” in which many of the viral non-structural proteins associate in addition to some host cell factors. Although the precise composition of the replication complex is unknown, its minimum composition must include the 3D^pol^ enzyme, primer 3B and viral RNA and *in vitro* studies also implicate a role for 3CD in the uridylation of the 3B primer protein. Each 3B peptide undergoes 3D^pol^-mediated uridylation on the third residue, which is tyrosine, to form VPg-pUpU (the primer for RNA synthesis) [40, 42, 43, 49–53]. Uridylation of 3B-containing precursors has also been demonstrated and the actual substrate for 3D^pol^-mediated uridylation has been postulated to be a higher-order 3B-containing precursor containing 3A and/or 3CD [6, 7, 53, 54].

FMDV has several distinct genetic features that distinguish it from other members of the *Picornaviridae* including three non-identical copies of 3B (termed 3B1, 3B2 and 3B3). Although the functional consequences for replication of these multiple copies of 3B are poorly understood, they are all present in equal amounts on encapsidated viral RNA molecules [41]. The presence of three copies of 3B is conserved in all but one natural isolate to date, suggesting a strong selective pressure for maintenance of all three copies in the field, although the driving force for this selection is not understood. In earlier studies, Falk *et al* concluded that although FMDV replication can occur with a single copy of 3B (e.g. 3B3), all three copies are required for maximum replication efficiency. However, they also found that viruses lacking 3B3 were not viable and proposed that this was due to aberrant processing at the novel 3B2/3C junction generated by deleting 3B3 [49]. Subsequently, Pacheco *et al* [55] and Arias *et al* [56] reported that viruses lacking both 3B1 and 3B2 were viable, with the former study reporting normal growth phenotypes in BHK-21 cells. Arias *et al* went on to suggest that although a virus with a single 3B had near normal RNA replication in BHK-21 cells, virus particle release was severely compromised [56]. Arias *et al* also reported that recombinant viruses with only 3B1 were non-viable but infectivity could be recovered by replacing the last four C-terminal residues of 3B1 with the corresponding residues of 3B3 (essentially generating a chimeric 3B1/3 molecule). These observations are in agreement with those of Falk *et al* and suggest that, as well as priming viral RNA (vRNA) synthesis, 3B3 has an additional function in replication that is mediated by the C-terminal residues. However, the precise nature of this role remains to be established.

The main goal of the current study was to determine the role(s) played by 3B3 in FMDV replication. Our initial studies confirmed the requirement for the wild-type sequence at the C-terminus of 3B3 for replication, which was independent of its role as a replication primer. Using a combination of pulse-chase labelling-studies and recombinant viruses we show that the C-terminal residues of 3B3 play a pivotal role in replication by controlling the rate of cleavage at the 3B3-3C boundary and downstream processing of 3CD. In addition, co-transfection recovery experiments show that 3B can be supplied for replication *in trans*. Thus, our results support a replication model where polyprotein processing is dynamically regulated to allow for the production of important processing intermediates as well as the mature proteins. Furthermore, our results reveal an important role for 3B3 in replication in addition to its role as a primer.

## Results

### Deletion of 3B3 is detrimental to FMDV replicon replication

Uniquely among the *Picornaviridae*, FMDV encodes three non-identical sequential copies of 3B, which serve as protein primers for replication. Previous studies using recombinant viruses have shown that, although no specific copy of 3B is essential for viability, residues within the C-terminal region of 3B3 are important for virus replication [49, 56]. Here, we have used an FMDV replicon containing the fluorescent reporter protein mCherry in place of the viral structural proteins [57, 58], with the aim of understanding the functional importance of 3B3 in FMDV replication. Monitoring the production of the mCherry reporter allows for real-time, indirect quantification of vRNA replication without the production of infectious virus.

**Fig 1:**
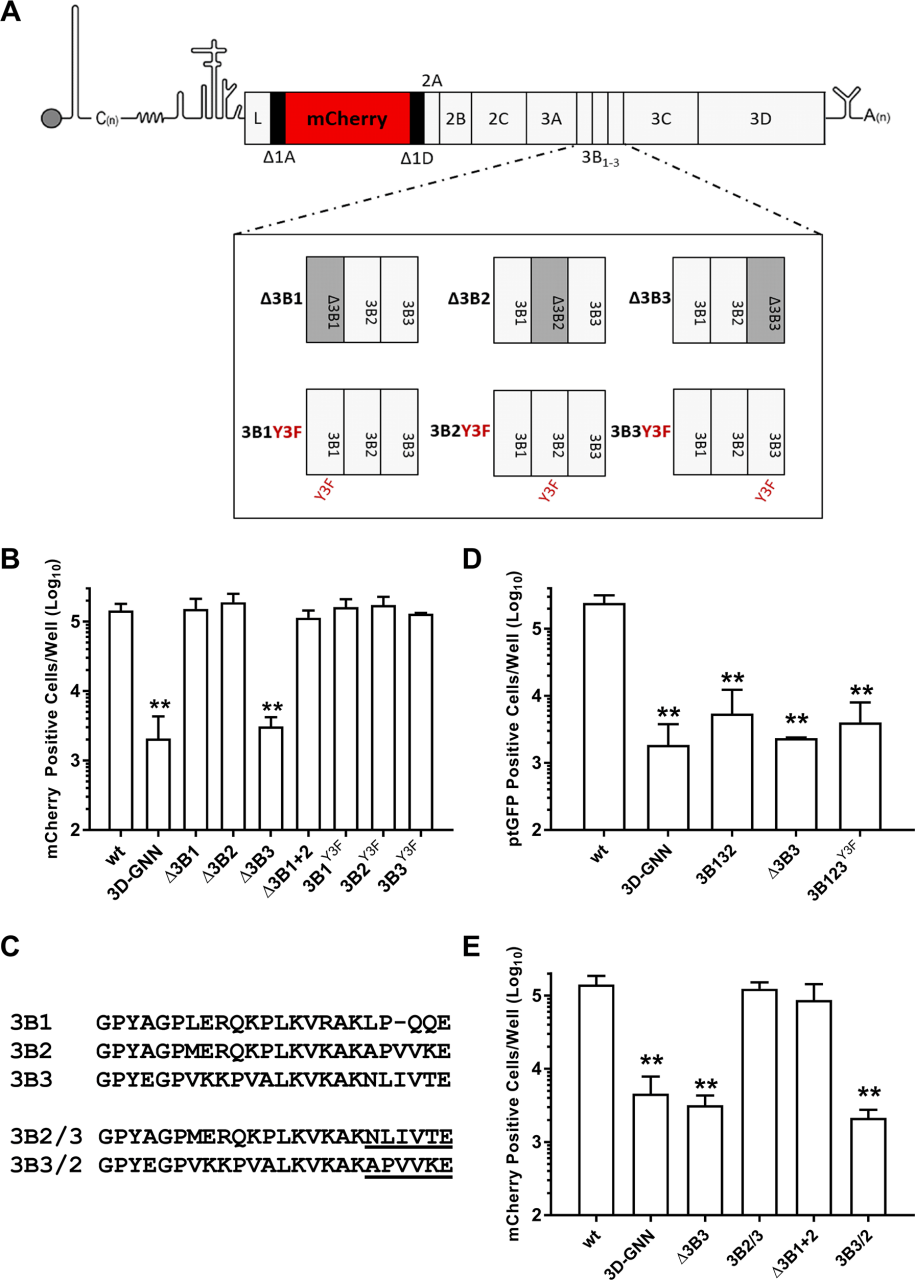
Residues at the 3B-3C boundary are important for replicon replication. **(A)** Schematic of the FMDV sub-genomic replicons bearing 3B deletions and tyrosine mutations used in this study. The 3B region is expanded for clarity. **(B)** BHK-21 cells seeded into 24 well plates were transfected with mCherry replicons bearing 3B deletions or mutations as well as wild-type (wt) and replication-defective polymerase mutant (3D^pol^-GNN) controls. Transfections were performed with replicons from which 3B1, 3B2 or 3B3 had been deleted (Δ3B1, Δ3B2, and Δ3B3, respectively), both 3B1 and 3B2 deleted in tandem (Δ3B1+2) or point-mutation to the uridylatable tyrosine of 3B1, 3B2 or 3B3 (3B1^Y3F^, 3B2^Y3F^ or 3B3^Y3F^, respectively). Expression of mCherry was monitored hourly over a 24 hour period. Data shown represent mean mCherry positive cells per well at 8 hours post-transfection. Significance compared to wild-type control (n = 3 ± SD, * = p<0.05, ** = p<0.01). **(C)** Alignment of the replicon 3B amino acid sequences and the chimeric 3B boundary mutations (3B2/3 and 3B3/2) showing the specific amino acid sequences of each mutation underlined. **(D)** BHK-21 cells seeded into 24 well plates were transfected with a ptGFP replicon with the positions of 3B2 and 3B3 exchanged (3B132) and expression of ptGFP monitored hourly over a 24 hour period. The wild-type, 3D^pol^-GNN, Δ3B3 and 3B123^Y3F^ constructs were included as controls. Data shown represent mean ptGFP positive cells per well at 8 hours post-transfection. **(E)** BHK-21 cells seeded into 24 well plates were transfected with mCherry replicons bearing chimeric 3B mutations and expression of mCherry monitored hourly over a 24 hour period. The wild-type, 3D^pol^-GNN, Δ3B1+2 and Δ3B3 constructs were included as controls. Data shown represent mean mCherry positive cells per well at 8 hours post-transfection. Significance compared to wild-type control (n = 3 ± SD, * = p<0.05, ** = p<0.01).

Initially, six replicons were generated with each of the individual copies of 3B either deleted (Δ3B1, Δ3B2 and Δ3B3), or rendered non-functional by the introduction of a point mutation (tyrosine to phenylalanine [Y3F]) at the tyrosine residue in position 3 (3B1^Y3F^, 3B2^Y3F^ and 3B3^Y3F^) (Fig 1A). This tyrosine serves as the acceptor site for 3D^pol^-mediated uridylation of 3B to generate the replication primer VPg-pUpU. Mutation to phenylalanine renders 3B unable to act as a primer for replication [40, 41, 43, 49, 52, 59]. Alongside, a seventh replicon was generated in which both 3B1 and 3B2 were deleted (Δ3B1+2) to determine if retaining only 3B3 was sufficient to support RNA replication. RNA transcripts were generated from the above replicons and transfected into BHK-21 cells. In addition, cells were transfected with RNA derived from a wild-type replicon and from a replication-defective replicon with mutations at the active site of 3D^pol^ (3D^pol^-GNN), which served as a control for translation from the input RNA. RNA replication was monitored by following fluorescence due to mCherry expression over a 24 hour period, with relative replication shown as the number of mCherry positive cells at 8 hours post-transfection, when the number of mCherry positive cells was maximal (S1 Fig). Our previous work has shown that identical results were obtained by measuring either total fluorescence or fluorescent cell numbers and the latter is shown here [60].

The results of these experiments are shown in Fig 1B. Consistent with previous studies [49, 55, 56], no significant differences were seen in the level of replication compared to the wild-type control upon deletion of 3B1 (Δ3B1) or 3B2 (Δ3B2), or both 3B1 and 3B2 together (Δ3B1+2). In contrast, deletion of 3B3 reduced reporter expression by approximately 100-fold, to a level comparable to the 3D^pol^-GNN replication-deficient control (i.e. to the level of input translation). In addition, there were also no significant differences in replication between the wild-type replicon and the replicons bearing individual non-uridylatable Y3F mutations in any of the 3B molecules. These observations agree with previous data and confirm that deletion of 3B3, but not 3B1 or 3B2, is detrimental for vRNA replication. However, mutations that render individual copies of 3B non-functional (including 3B3) do not inhibit replication.

### Residues at the C-terminus of 3B3 are important for FMDV replication

The observations above showed that deletion of 3B3 prevented vRNA replication whilst an inactivating mutation in 3B3 showed no decrease in replication, suggesting that 3B3 has a key functional role additional to its role as a replication primer. The three copies of 3B show greater sequence similarities in the N-terminal regions than the C-terminal regions, which show little sequence similarity between molecules (Fig 1C). The previous study by Arias *et al* implicated the residues at the C-terminus of 3B3 as being important for FMDV replication, as a mutant virus with only 3B1 required exchange of a minimum of four amino acids from the C-terminus of 3B3 for viability. In addition, studies with other members of the *Picornaviridae* have highlighted the importance of the 3B-3C cleavage boundary [54, 61, 62].

To extend these observations, we investigated the importance of the sequence at the C-terminus of 3B3 in FMDV vRNA replication. Initially, we determined if the location of 3B3 within the polyprotein is important for replication by constructing a replicon where the positions of 3B2 and 3B3 were exchanged (3B132). Replication of vRNA was assessed in BHK-21 cells alongside the wild-type and 3D^pol^-GNN controls. We also constructed an additional control, where all three copies of 3B incorporated the defective Y3F mutation (3B123^Y3F^). The results of this experiment (Fig 1D) show that exchange of the positions of 3B2 and 3B3 resulted in a greater than 50-fold reduction in replication compared to the wild-type control. Indeed, there is little difference between the reporter gene expression from this RNA and the 3D^pol^-GNN, Δ3B3 or 3B123^Y3F^ replicons. Therefore, the relative position of 3B3 within the P3 polyprotein is critically important for replication, which implies that the specific sequence at the 3B-3C boundary determines replicative competence.

To further investigate the role of the 3B3 C-terminal residues in replication, two additional replicons were generated. The first, termed 3B2/3, had an internal deletion between residue 18 of 3B2 and residue 18 of 3B3. This resulted in a replicon which had two copies of 3B; a wild-type 3B1 and a chimera formed from residues 1–18 of 3B2 fused to residues 19–24 of 3B3 (Fig 1C). The second construct, termed 3B3/2, retained 3 copies of 3B but had the last six residues of 3B3 changed to those of 3B2 (Fig 1C). Replication was assayed, alongside the Δ3B1+2 and Δ3B3 replicons and the wild-type and 3D^pol^-GNN controls (Fig 1E). Consistent with our previous observations (Fig 1B), deletion of 3B3 reduced reporter expression to a similar level to the 3D^pol^-GNN control. However, substitution of the final 6 residues of 3B2 for those of 3B3 (3B2/3) completely restored replication to wild-type levels. Furthermore, consistent with the data shown in Fig 1B, the replicon with both 3B1 and 3B2 deleted (Δ3B1+2) showed levels of replication equivalent to the wild-type replicon. However, substitution of the final residues of 3B3 for those of 3B2 (3B3/2) reduced replication to a level similar to that of the 3D^pol^-GNN control. Thus, these experiments confirm that the C-terminal residues of 3B3 play a key role in FMDV replication that is independent of the role of 3B3 as a primer.

### Mutations to the C-terminus of 3B3 prevent downstream processing of 3CD

**Fig 2:**
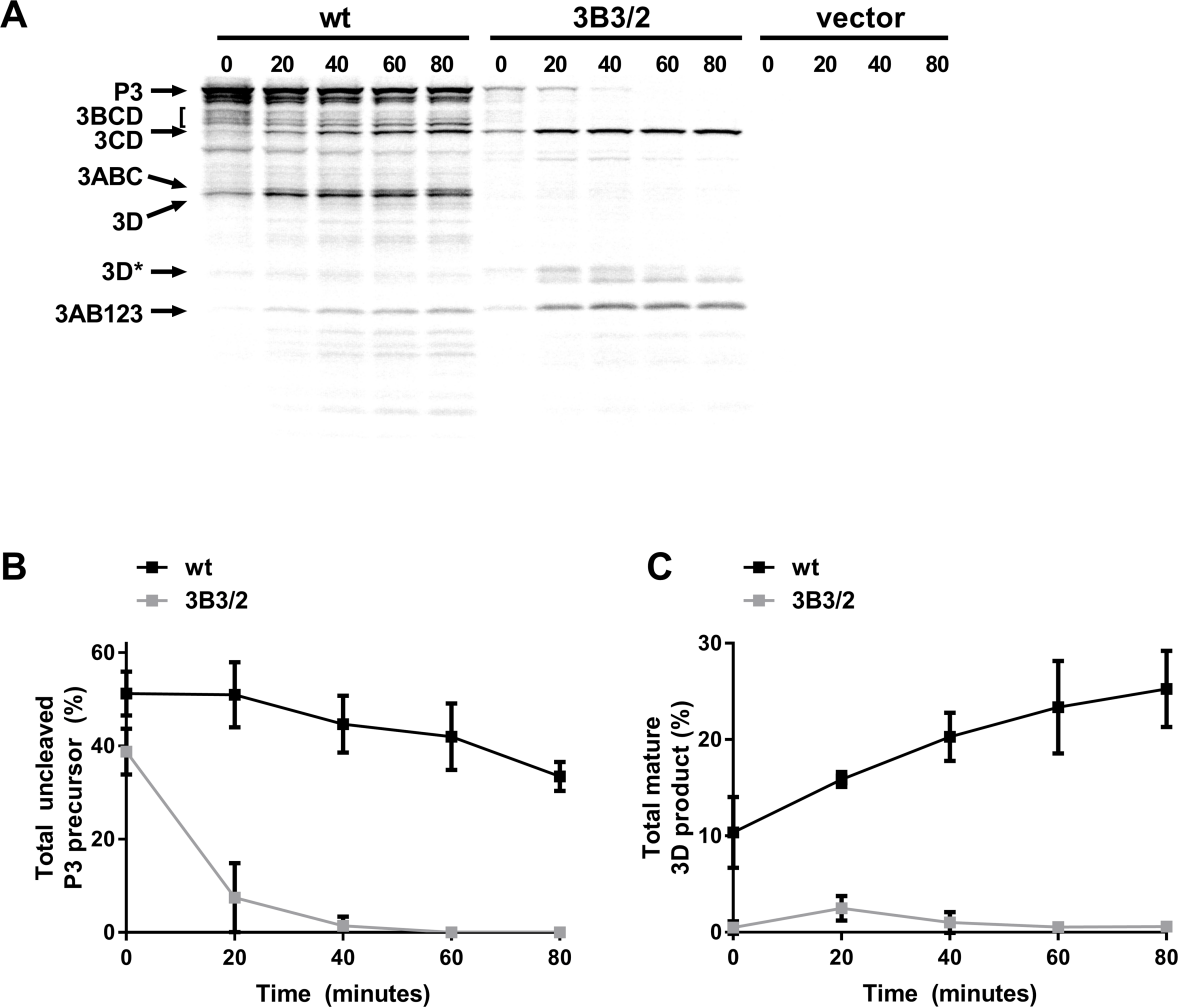
Mutations at the 3B-3C boundary disrupt P3 polyprotein processing. Plasmid constructs expressing wild-type FMDV P3 or the 3B3/2 chimeric mutant polyprotein were used to assemble coupled transcription/translation reactions with [^35^S] labelled methionine. Reactions were incubated for 40 minutes before addition of excess unlabelled methionine/cysteine, samples were taken at regular intervals and reaction stopped by the addition of 2 x Laemmli buffer. **(A)** Proteins were separated on 12% SDS-PAGE and visualised by autoradiography. The positions of FMDV protein products are indicated by arrows, the 3D* product represents a degradation or cleavage product of 3D^pol^ (as confirmed by Western blot, see S2 Fig). Control reactions were assembled using empty expression vector alone. The proportion of uncleaved P3 **(B)** and 3D^pol^ **(C)** product was quantified as a percentage of the total 3D^pol^ containing products (n = 2 ± SD).

Taken together, our observations are in agreement with previous reports that implicate the residues at the C-terminus of 3B3 as important for FMDV replication. These data also suggest that the position of 3B3 within P3 and sequence conservation at the 3BC boundary are critical. To investigate the mechanism behind this, we determined the effect of changing the 3B-3C boundary sequence on processing of the P3 precursor. This was done using an *in vitro* coupled transcription/translation assay as the replication-defective phenotype of the 3B2/3 mutation made a direct analysis of P3 processing using replicons not possible. T7 expression constructs were generated to express either the wild-type FMDV P3 polyprotein or P3 containing the 3B3/2 mutation (i.e. with wild-type 3B1 and 3B2 plus a chimeric 3B3, 3B3/2). These constructs were used for [^35^S] methionine pulse-chase labelling in *in vitro* transcription/translation, with samples harvested at regular intervals and the protein products analysed by SDS-PAGE (Fig 2A). The identity of each band was subsequently confirmed by Western blot analysis (S2 Fig). The proportions of unprocessed P3 and 3D^pol^ were quantified by phosphor-imaging as percentages of the total 3D^pol^-containing products. (Fig 2B and 2C). For the wild-type precursor, unprocessed P3 could clearly be detected after 0 minutes of chase, in addition to the precursors 3ABC, 3BCD and 3CD and fully processed 3D^pol^. Over time an accumulation of 3CD and 3D^pol^ was seen with a concomitant reduction of the 3BCD and P3 precursors. At the later time points, 3AB123 could be readily detected. In comparison to the wild-type construct, the P3 precursor containing the 3B3/2 mutation produced less P3 precursor, but greater amounts of 3AB123 and 3CD, which could be detected even at the early time points. Despite a clear accumulation of 3CD, little or no 3D^pol^ was detected at any of the time points including at 80 minutes post-chase. In addition, little 3BCD was detected even at 0 minutes with the 3B3/2 mutant. The P3 polyprotein and an additional doublet (termed 3D*) was observed at approximately 35 kDa that reacted with an anti-3D^pol^ antibody. This doublet likely corresponds to non-specific or incorrect proteolysis of 3CD, possibly similar to that observed in studies with PV [48, 63]. Quantification of unprocessed P3 and 3D^pol^ confirmed the observations that the initial processing of P3 was faster for the 3B3/2 mutant than the wild-type P3 polyprotein. Furthermore, the 3B3/2 containing precursor generated virtually no 3D^pol^ (Fig 2B and 2C). These experiments therefore suggest that the mutations introduced at the C-terminus of 3B3 to generate 3B3/2 interfered with the normal processing dynamics by increasing the processing efficiency at the 3B-3C boundary, which in turn resulted in a defect in 3CD processing that prevented the generation of fully processed 3C^pro^ and 3D^pol^. The total amount of [^35^S] methionine incorporation with the 3B3/2 mutant P3 precursor was somewhat less than that for the wild-type in this assay, possibly due to differences in translational efficiency of the two constructs. Furthermore, accurate quantification of total incorporation is not possible in these experiments due to the differences in the processed proteins produced by these constructs.

### Mutations at the C-terminus of 3B3 prevent complementation of replication-defective 3D^pol^ mutations but not 3B mutations

Processing of 3AB may be required to provide 3B as a primer for replication. In addition, although for PV 3CD has been shown to be a functional protease with a different specificity to the 3C^pro^, it is inactive as a polymerase and 3CD cleavage would be required to produce a functional 3D^pol^. Thus, organised processing of intermediate protein products is likely to be critical for viral replication. Our data, derived from *in vitro* transcription/translation experiments, show that increasing the rate of cleavage at the 3B-3C boundary interferes with correct P3 processing and results in the accumulation of 3AB123 and 3CD intermediates which are not further processed, suggesting that 3CD is either inactive as a protease or cannot recognise the cleavage boundaries present in these intermediate proteins. However, these experiments were in the absence of active FMDV replication, and we wished to determine if increasing the rate of 3B-3C cleavage prevents the production of functional 3B or 3D^pol^ in the context of RNA replication. To this end, we employed a *trans*-complementation system we described recently [60]. This system involves simultaneous co-transfection of two replication-defective replicons that express different fluorescent reporter genes, allowing for their replication to be assayed separately. Co-transfection of the two replicons enables exchanges of viral non-structural proteins to facilitate replication of one or both of the input replicons. In this way it is possible to probe whether mutations introduced into one replicon can be compensated by the wildtype protein supplied by another *in trans*.

**Fig 3:**
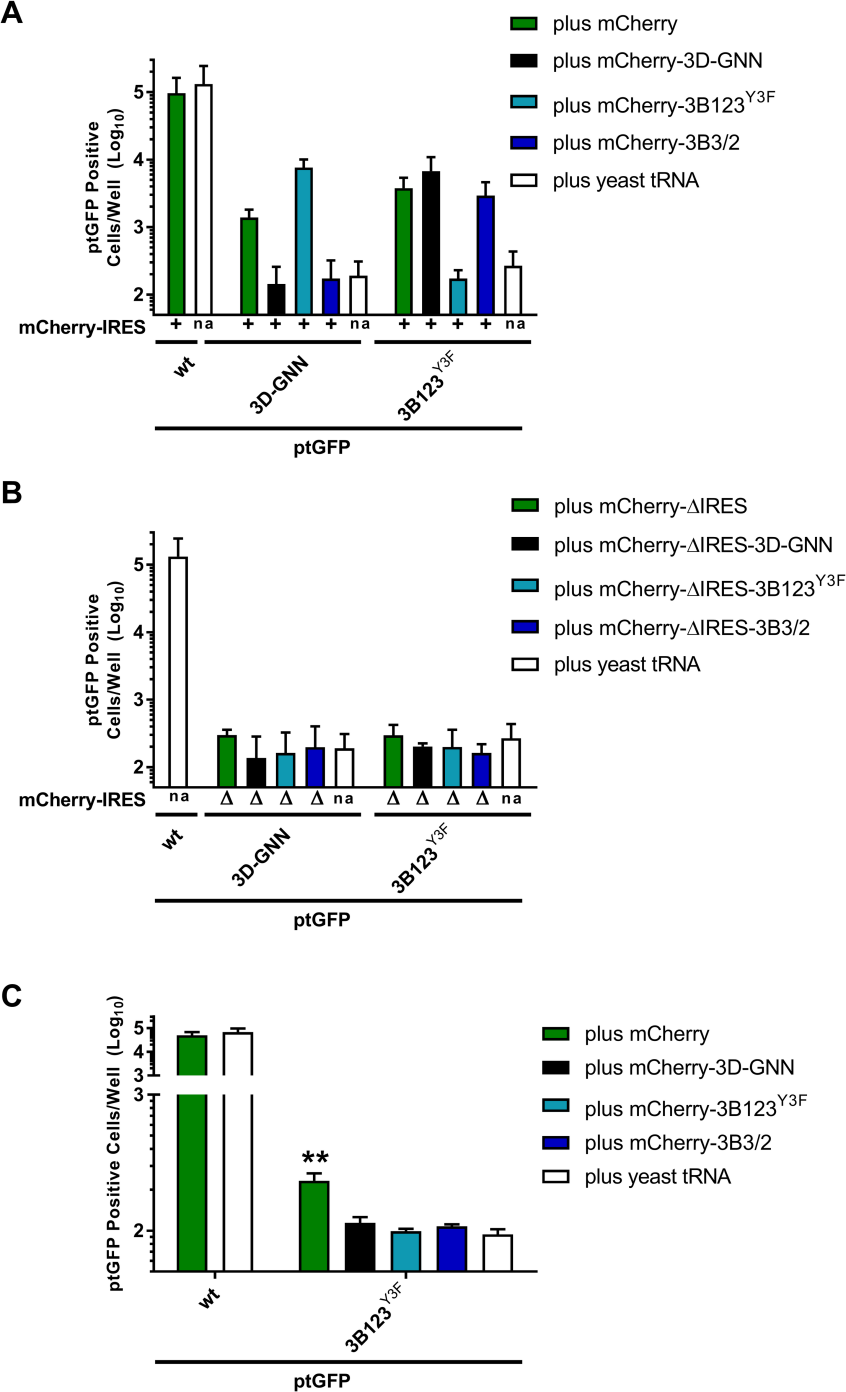
Mutations at the C-terminus of 3B3 prevent complementation of 3D^pol^ mutations but not 3B mutations *in trans*. **(A)** BHK-21 cells seeded into 24-well plates were co-transfected with mCherry replicons bearing replication-defective 3B or 3D^pol^ mutations or controls and wild-type ptGFP, ptGFP-3B123^Y3F^ or ptGFP-3D^pol^-GNN replicon. In **(A)** all the mCherry replicons contained a full IRES (+). In **(B)** the mCherry replicons contained a deletion of the entire IRES (Δ), in addition to the indicated non-structural protein mutation (3D^pol^-GNN, 3B123^Y3F^, 3B3/2). Co-transfections were performed with yeast tRNA (bars labelled ‘na’) as a negative control (i.e. no complementation) in both experiments. Expression of ptGFP is shown representing mean positive cells per well at 8 hours post transfection (n = 3, ± SD). For clarity the statistics were excluded from the figure. The mCherry data is shown in S3 Fig. In **(C)**, after the initial the co-transfection, total RNA was harvested and replicons RNA purified by oligo(dT) precipitation. 100 ng of total oligo(dT) purified RNA was re-transfected into BHK-21 cells seeded into 24-well plates and expression of ptGFP and mCherry were monitored hourly. Data shows mean ptGFP positive cells per well at 8 hours post transfection. Significance compared to plus yeast tRNA negative control. (n = 5 ± SD, * = p<0.05, ** = p<0.01).

**Fig 4:**
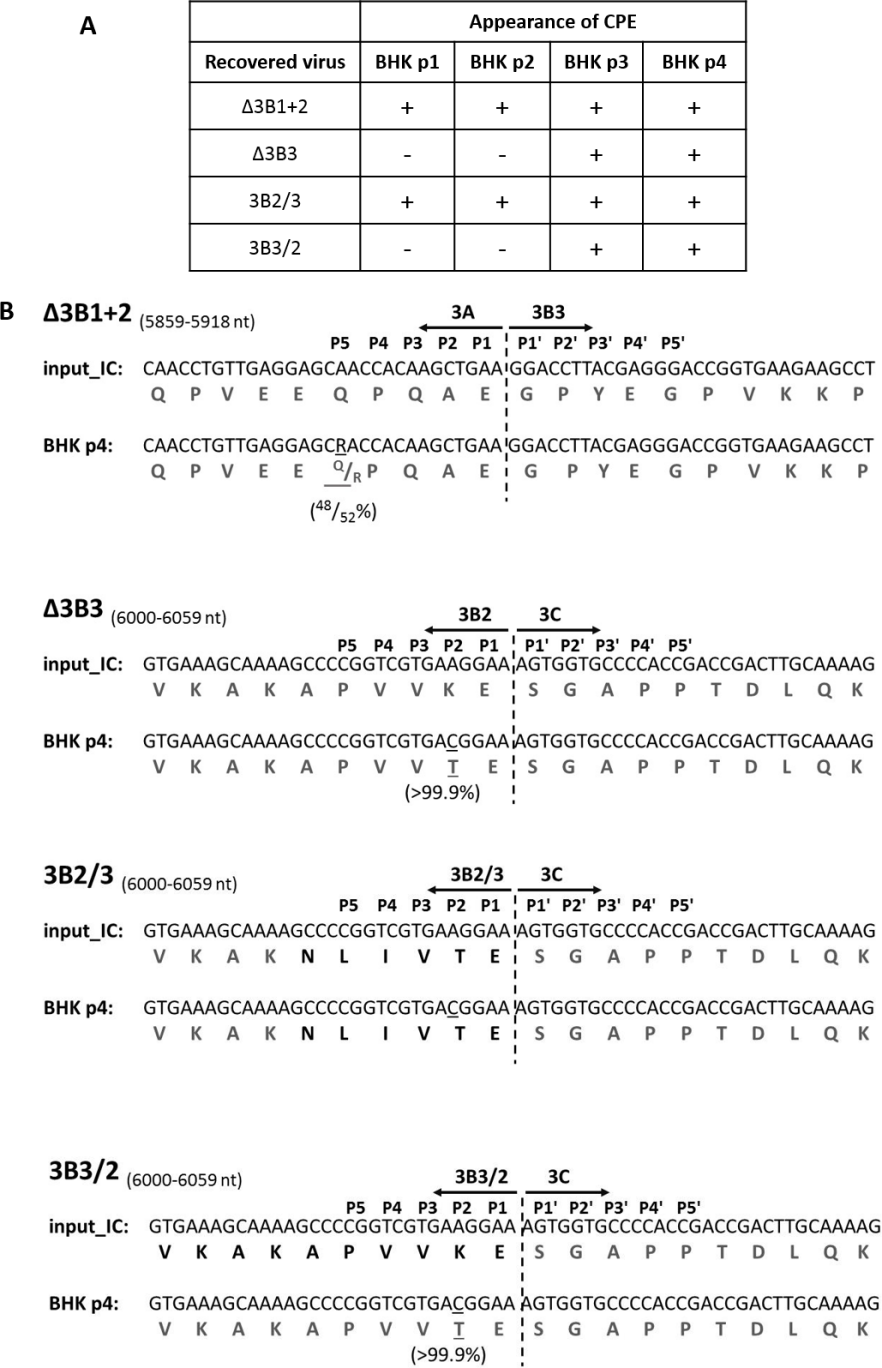
O1 Kaufbeuren virus containing Δ3B3 mutation changes 3B2-3C cleavage site to resemble 3B3-3C boundary. **(A)** Δ3B1+2, Δ3B3, 3B2/3 and 3B3/2 replicons were converted into infectious copy (IC) constructs by insertion of the capsid encoding region of O1 Kaufbeuren (O1K) FMDV isolate. RNA transcribed from IC constructs was transfected into BHK-21 cells and virus was recovered by freeze/thawing. Recovered viruses were blindly passaged up to four times in BHK-21 cells (BHK p1-4); each time infected cells were monitored for appearance of CPE. **(B)** All four viruses which had undergone four passages (BHK p4) were sequenced using Illumina MiSeq platform. Consensus and subconsensus sequences of recovered viruses (BHK p4) were compared to the input IC construct (input_IC). Part of sequences (nucleotide in black and amino acid in dark grey) where changes in recovered viruses were observed in comparison to input IC construct are shown. Mutated nucleotides/amino acids are underlined; percentage of virus population bearing the mutation is shown underneath in brackets. Dashed line indicates the scissile bond dipeptide cleavage site with the flanking boundary residues indicated and designated P5 to P5' and according to the standard nomenclature. Nucleotide range shown in brackets indicates position of the presented sequence in relation to genome of recovered viruses.

We utilised this system to determine if the 3B-3C processing defect inhibited complementation of replicons with mutations in 3B or 3D^pol^. Replication-defective mCherry constructs bearing the triple 3B Y3F mutation (3B123^Y3F^), 3B3/2 mutation or the catalytic 3D^pol^ mutation (3D^pol^-GNN) were co-transfected with ptGFP expressing replicons containing either the replication-defective triple 3B Y3F mutation (3B123^Y3F^) or 3D^pol^-GNN. Alongside, co-transfections were performed with the wild-type mCherry or ptGFP constructs, to check for possible dominant-negative effects, and with yeast tRNA as a negative control (i.e. no complementation). Replication of both mCherry and ptGFP replicons was monitored by fluorescent reporter gene expression and the number of cells expressing ptGFP quantified at 8 hours post-transfection (Fig 3A). Replication of the wild-type ptGFP replicon did not significantly change upon co-transfection with any of the mCherry replicons tested when compared to co-transfection with the yeast tRNA control, suggesting no dominant negative effects on replication (for clarity only the wild-type ptGFP with wild-type mCherry data are shown on Figs 4A and S3). Replication of the ptGFP-3B123^Y3F^ replicon was significantly recovered by all the mCherry replicons tested except the mCherry-3B123^Y3F^ control, showing that 3B can be efficiently supplied *in trans*. In contrast, although the ptGFP-3D^pol^-GNN replicon was recovered by the wild-type mCherry or mCherry-3B123^Y3F^ constructs (which produce a functional 3D^pol^), replication could not be recovered by mCherry-3B3/2. These data are therefore consistent with the *in vitro* data (Fig 2), showing that the 3B3/2 construct is unable to provide 3D^pol^ and therefore cannot function *in trans*.

Recombination in picornaviruses is well documented and it is important for interpretation of the above experiments to elucidate whether the observed rescue of ptGFP is due to complementation and/or recombination. Recent studies have described recombination in picornaviruses occurring at low levels in the absence of viral protein expression (for example, mediated by cellular RNA ligases etc; [64–66]), so called ‘non-replicative recombination’. It was possible that such a mechanism was responsible for the recovery observed in our experiments (i.e. recombination between ptGFP and mCherry replicons in the absence of protein expression leading to wild-type replicons). Therefore as an additional control, the experiments above were repeated with the same mCherry constructs but lacking the IRES. In this experiment no recovery was observed, indicating that complementation requires translation of both replicons and suggesting no ‘non-replicative recombination’ occurred in this system (Fig 3B).

In addition, we undertook a novel RNA passaging experiment. Following co-transfection of the potentially complementing replicon RNAs and allowing replication to occur, total cell RNA was extracted, mRNAs (including replicon RNAs) purified by oligo(dT) affinity selection and re-transfected into naïve BHK-21 cells. Due to the relative inefficiency of complementation, only genomes that have recombined to form replication competent genomes (in the initial co-transfection) will replicate when passaged onto naïve cells. For clarity only a subset of the data is shown (Fig 3C). The wild-type ptGFP replicon could be passaged successfully alone or after the initial co-transfection with any of the mCherry replicons. Furthermore, the ptGFP-3B123^Y3F^ replicon could be successfully passaged at a low level following initial co-transfection with the wild-type mCherry helper replicon, indicating that some recombination occurs following co-transfection. Therefore, the recovery of ptGFP-3B123^Y3F^ by the wild-type helper replicon is likely to be partially due to recombination. However, the ptGFP-3B123^Y3F^ replicon could not be passaged following initial co-transfection with either the mCherry-3D^pol^-GNN or mCherry-3B3/2 replicons, despite the significant levels of recovery observed in the initial co-transfection (Fig 3A). Together the results suggest that the recovery of replication seen in the above experiments (Fig 3A) is not due to either replicative or non-replicative recombination but results from *trans*-complementation by 3B. These replicon experiments suggest that, in addition to its role as a primer peptide for RNA replication, sequence elements within 3B3 serve to modulate precursor polyprotein processing to release functional 3D^pol^. The residues at the C-terminus of 3B3 (i.e. at the 3B-3C boundary) appear to control the rate of processing at this junction, which in turn influences the supply *in trans* of catalytically active 3D^pol^ and functional 3B peptides to support replication.

### FMDV infectivity does not require a specific copy of 3B

In order to determine whether the results obtained with replicons are replicated in virus infections, we first tested our conclusion that there is no requirement for a specific copy of 3B for FMDV RNA replication using recombinant viruses. The Δ3B1+2, Δ3B3, 3B2/3 and 3B3/2 mutations were introduced into an FMDV O_1_ K infectious copy plasmid and viruses recovered by transfection of BHK-21 cells with *in vitro* transcribed RNA. Viruses were then blind-passaged 4 times (P1-4) on BHK-21 cells and the cells monitored for the appearance of cytopathic effect (CPE) (Fig 4A).

For the Δ3B1+2 and 3B2/3 transcripts, CPE was detectable at the first passage (P1) in BHK-21 cells. In contrast, CPE was not evident for the Δ3B3 and 3B3/2 transcripts until passage 3 (Fig 4A). The delayed appearance of CPE is often indicative of the need to acquire compensatory mutations to facilitate efficient virus replication. To identify the presence of such additional mutations, RNA was extracted from viruses recovered at P4 and sequenced using an Illumina MiSeq platform. The consensus and sub-consensus sequences were compared to the corresponding input RNA (Fig 4B). The virus isolated from the Δ3B1+2 transfection maintained the deletions of 3B1 and 3B2. However, approximately 52% of the virus sequences contained a nucleotide substitution which resulted in a Q>R mutation near the C-terminus of 3A, at the P5 position within the 3A-3B cleavage site (the P5 to P5′ residues are indicated on Fig 4B). No sequence changes were observed in the virus recovered from the 3B2/3 transfections and >99.9% of the recovered sequences were identical to the input genome. The virus derived from the Δ3B3 transfection maintained the deletion of 3B3 but >99.9% of the sequences contained an additional mutation (A to C) that resulted in a K>T change near the C-terminus of 3B2, at the P2 position within the 3B-3C cleavage site (Fig 4B). The same mutation resulting in a K>T change was also observed in the virus derived from the 3B3/2 transfection, which otherwise retained the sequence of the input RNA.

These results confirm that there is no requirement for a specific 3B molecule for FMDV replication and infection and that each of the three peptides can function as a primer for RNA synthesis. In addition, the observations that recovery of infectious virus from the transcripts with the Δ3B3 or the 3B3/2 mutations requires an additional compensatory mutation within the 3B-3C cleavage site, further implicates the sequence at the 3B-3C boundary as of special importance for FMDV replication.

### Compensatory mutations at the 3B-3C boundary restore FMDV replicon replication

**Fig 5:**
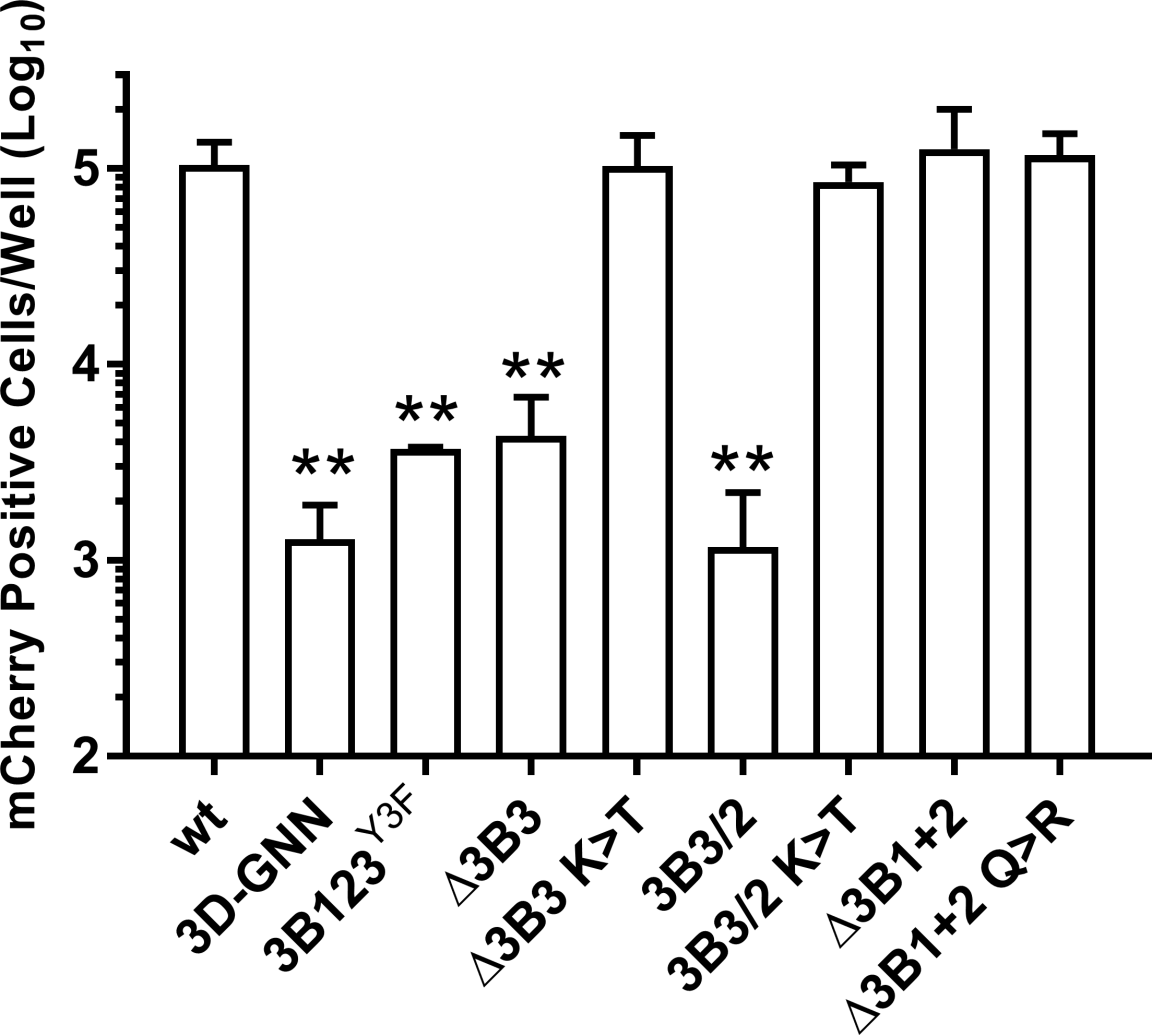
A single reversion mutation at the 3B-3C boundary restores replicon replication. BHK-21 cells seeded into 24 well plates were transfected with mCherry replicons bearing 3B mutations as indicated, alongside wild-type (wt) and replication-defective polymerase mutant (3D^pol^-GNN) controls, and expression of mCherry monitored hourly over a 24 hour period. Data shown represents mean mCherry positive cells per well at 8 hours post-transfection. Significance compared to wild-type control (n = 3 ± SD, * = p<0.05, ** = p<0.01).

The K>T mutation identified above in the recovered Δ3B3 and 3B3/2 recombinant viruses (Fig 4) restores the amino acid at P2 within the 3B3-3C cleavage site to the wild-type residue. To confirm that this single change is sufficient to rescue replication of the parental Δ3B3 and 3B3/2 replicons, the K>T mutation was introduced into the 3B3/2 and Δ3B3 mCherry replicons, to generate 3B3/2 K>T and Δ3B3 K>T, respectively. Replication of vRNA was assayed in BHK-21 cells alongside the control replicons, as before (Fig 5). Consistent with our previous observations, deletion of 3B3 (Δ3B3) or the 3B3/2 mutation (3B3/2) dramatically reduced replication compared to the wild-type control. However, replication of the Δ3B3 K>T and 3B3/2 K>T mutants was completely restored to wild-type levels.

### Compensatory 3B-3C boundary mutations restore correct P3 processing

**Fig 6:**
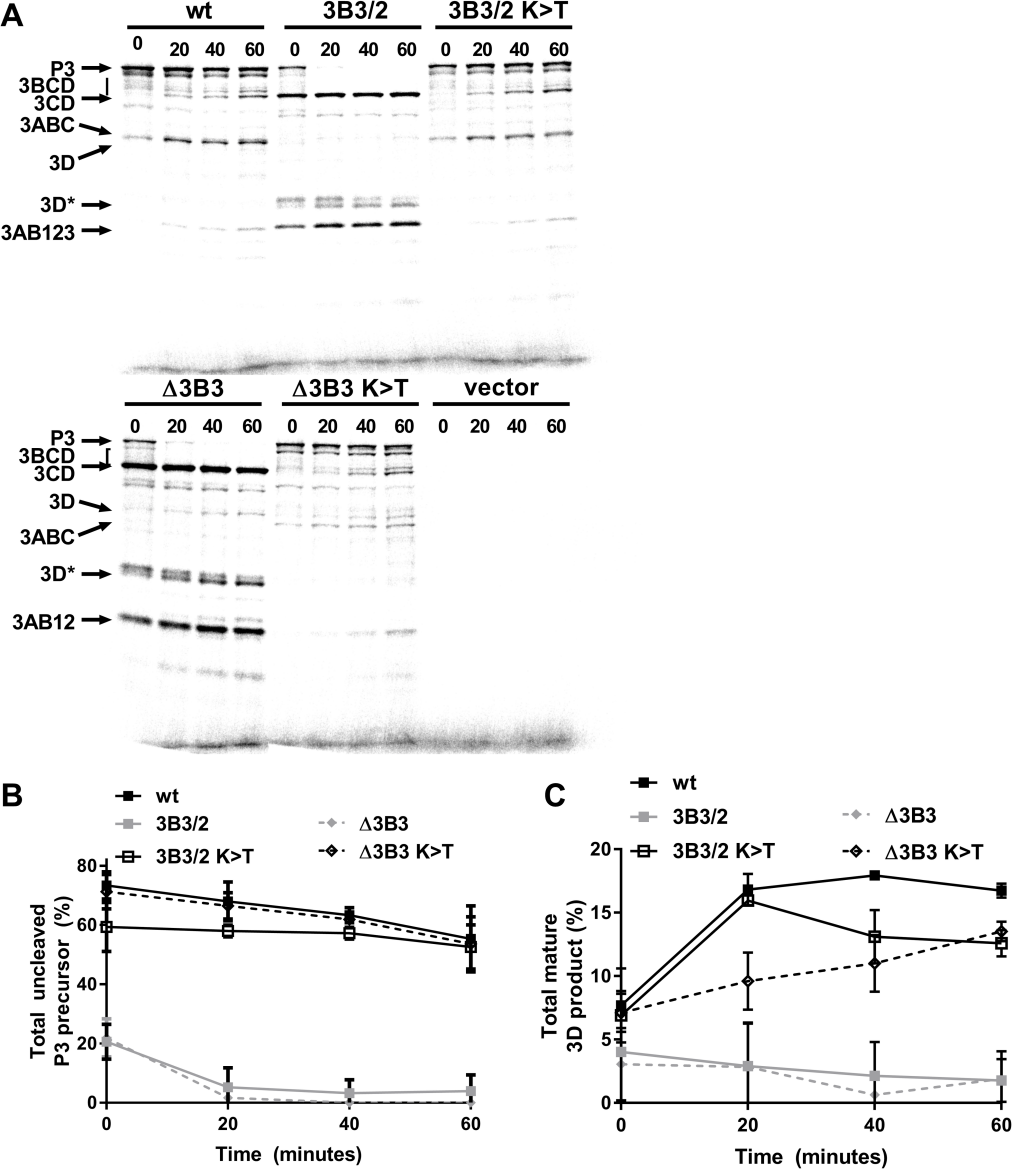
Reversion mutations at the 3B-3C boundary restore P3 polyprotein processing. Plasmid constructs expressing wild-type or mutant P3 polyprotein were used to assemble coupled transcription/translation reactions with [^35^S] labelled methionine. Reactions were incubated for 40 minutes before addition of excess unlabelled methionine/cysteine, samples taken at regular intervals and reactions stopped by the addition of 2 x Laemmli buffer. **(A)** Proteins were separated on 12% SDS-PAGE and visualised by autoradiography. The positions of FMDV protein products are indicated by arrows. Control reactions were assembled using empty expression vector alone. The proportion of uncleaved P3 **(B)** and 3D^pol^ **(C)** product was quantified as a percentage of the total 3D^pol^ containing products (n = 2 ± SD).

The K>T mutation at the P2 position of the 3B3-3C cleavage site restored replication to both the Δ3B3 and 3B3/2 replicons. We therefore anticipated that this mutation might restore normal P3 processing. To test this, T7 expression constructs were generated to express the 3B3/2 K>T or Δ3B3 K>T mutants within P3 polyproteins and used in the *in vitro* coupled transcription and translation assay described above (Fig 6A). The proportion of unprocessed P3 and 3D^pol^ were quantified as before as a percentage of the total 3D^pol^ containing products (Fig 6B and 6C).

The wild-type precursor gave an identical processing pattern to that shown in Fig 3. Unprocessed P3 was clearly evident at early time points and was processed over time with an accumulation of 3CD and 3D^pol^. Similarly, the 3B3/2 mutant polyprotein demonstrated a near identical pattern to before and showed little detectable P3 precursor, an increased rate of 3B-3C cleavage, an accumulation of the 3CD precursor and virtually no 3D^pol^ protein. In addition, Fig 6A also shows that the Δ3B3 mutant polyprotein had similar processing defects to the 3B3/2 mutant as P3 was rapidly processed to the 3AB12 and 3CD precursors but virtually no 3D^pol^ was generated. However, introducing the K>T mutation into either of the 3B3/2 or Δ3B3 constructs (3B3/2 K>T and Δ3B3 K>T) restored P3 processing to better resemble that for the wild-type polyprotein. These data are consistent with the conclusion that the rate of processing at the 3B-3C boundary is tightly regulated and that the timing of cleavage is critically important for releasing catalytically active 3D^pol^ from the polyprotein.

Taken together, these data support our conclusion that the precise-timing of the cleavage between 3B and 3C^pro^ is essential for generating functional 3D^pol^ for viral replication and suggest that there are multiple, mutually-exclusive, polyprotein processing pathways which determine when 3CD is cleaved to release active polymerase.

## Discussion

In this study, we have demonstrated how a single, small viral protein, 3B3, has multiple functions during FMDV infection by serving both as a primer for RNA replication and to direct processing of the P3 polyprotein thereby controlling the release of the viral polymerase for replication.

In agreement with previous studies, we demonstrate with replicons and infectious virus that replication can proceed following deletion of two of the three tandem copies of the primer peptide, 3B, leaving just 3B3 (i.e. Δ3B1+2) [49, 55, 56]. Furthermore, our findings demonstrated that deletion of 3B3 alone (Δ3B3) was detrimental to replication, whereas mutating the uridylated tyrosine residue of 3B3 (3B3^Y3F^) had no significant effect on replicon replication. This difference was unique to 3B3 and deletion or inactivation of 3B1 or 3B2 (i.e. Δ3B1, Δ3B2, 3B1^Y3F^ or 3B2^Y3F^) had little effect on replication, largely in agreement with aforementioned studies. This implies that 3B3 has a special significance in FMDV replication independent of its role as a primer for RNA synthesis [53, 56, 67].

Arias *et al* identified that chimeric 3B1/3 molecules can compensate for 3B3 deletions [56]. Therefore, we generated a replicon in which 3B3 was deleted and the final six C-terminal residues of 3B2 were exchanged for those of 3B3 (3B2/3). Alongside, a replicon was constructed in which the final 6 residues of 3B3 were replaced for those of 3B2 (3B3/2). In agreement with Arias *et al*, the replicon where the 3B-3C junction was maintained (3B2/3) was replication-competent. Furthermore, the chimeric replicon (3B3/2) in which a 3B2-3C junction was introduced was replication-defective. Both pieces of evidence support the hypothesis that a specific sequence at the C-terminus of 3B3, at the 3B-3C boundary, is essential for FMDV replication. When either the 3B3/2 or Δ3B3 mutations were introduced into a full genome length clone, virus was recovered after serial blind passage. However, although both of these viruses maintained the original mutations, they had acquired a compensatory mutation at the 3B-3C boundary (reversion of the P2 residue from lysine to threonine). This provides further evidence for specific sequence requirements at the C-terminus of 3B3 for replication. Furthermore, since virus was recoverable with 3B3 deleted (albeit with a compensatory mutation), it would suggest that there is no stringent requirement for the entire 3B3 sequence *per se* for virion production.

Introduction of mutations at the cleavage boundaries within polyproteins is known to have effects on the rate of boundary cleavage [54, 68, 69]. For picornaviruses there is evidence from *in vitro* studies that the rate of processing can be largely influenced by the sequences at boundary junctions [38, 70, 71]. Hence, we chose to investigate the effect of the 3B-3C boundary mutations used in this study on processing of the P3 polyprotein precursor. Earlier reports have suggested that deletion of 3B3 produces a chimeric 3B2-3C junction which results in defective P3 processing, possibly by preventing 3B-3C cleavage [49]. However, we have now shown that the replication-defective 3B3/2 mutation did not decrease 3B-3C cleavage, but actually increased it. Our data also show that increasing 3B-3C cleavage unexpectedly rendered the 3C-3D junction non-cleavable, despite there being no mutations in either of these proteins. In agreement with this, the reversion at the P2 residue of the 3B-3C boundary identified from infectious virus (i.e. 3B3/2 K>T and Δ3B3 K>T), reduced the rate of 3BC cleavage and restored normal polyprotein processing and replicon replication.

Our observations therefore imply that 3CD cannot undergo auto-proteolysis to provide 3D^pol^. Although PV 3CD is proteolytically-active it cannot function as a polymerase [12, 27–29, 46–48]. We therefore investigated the effect of increasing the rate of 3B-3C cleavage (so preventing release of 3D^pol^ from the polyprotein) on replication of the FMDV replicon. To do this we implemented a genetic complementation system which we have recently described for probing the recovery of replication-defective FMDV replicons. In our complementation experiments, 3B could be supplied *in trans*. However, replicons with a mutant 3B-3C boundary (3B3/2) could not provide help *in trans* to recover replication of replicons with a mutation in 3D^pol^ (3D^pol^-GNN), but could rescue replicons bearing mutations to the tyrosine residues of 3B (3B123^Y3F^). Thus, our intracellular complementation results support the conclusion derived from *in vitro* data that increasing the rate of cleavage at the 3B-3C boundary results in the production of 3CD, which cannot be processed further to release functional 3D^pol^.

It is important to consider additional potential consequences of regulating the rate of 3B-3C cleavage on P3 processing. Here we show that increasing the rate of cleavage at the 3B-3C site dramatically decreases the amount of the unprocessed P3 precursor as well as the 3BCD and 3ABC processing intermediates. For PV, the P3 precursor has been reported to be part of a large multiprotein complex that is required for replication and can serve as a source of 3D^pol^ and 3B in complementation studies [16]. Other studies with PV and FMDV have also implicated larger 3B-containing precursors (such as 3ABC and 3BCD) as having important roles in viral replication [53, 54, 67, 69]. It is therefore possible that the replication defect introduced by increasing the rate of processing at the 3B-3C boundary (e.g. the 3B3/2 mutation) is in part the result of the reduced abundance of unprocessed P3 and/or the 3ABC and 3BCD precursors. This could also explain why the 3B3/2 mutation was unrecoverable *in trans* by helper replicons (unlike other 3B mutations such as 3B123^Y3F^).

It is clear from our results that the precursor protein, P3, can be processed by at least two mutually exclusive cleavage pathways. Considering this observation, it is likely the P3 precursor can be differentially processed *in cis* and *in trans*. Deciphering the different *cis/trans* cleavage pathways is important for understanding how processing of the polyprotein is temporally regulated. From our observations it appears likely that initial processing of the 3C-3D and 3B3-3C boundaries of the wild-type precursor are performed *in cis*, helping to explain why the 3B3-3C boundary is so sequence-sensitive. Thus, mutations at the 3B3-3C boundary that favour faster *cis* cleavage at this junction than at the 3C-3D junction lead to an accumulation of un-processed, non-active 3CD. Alternatively, if the 3C-3D boundary is processed *in cis* in the normal P3 precursor, (followed by *cis* processing at 3B3-3C boundary), the 3B3/2 mutation may favour *trans*-mediated cleavage at this boundary, thus inhibiting subsequent *cis*-mediated processing of 3CD. Experiments using an *in vitro trans* cleavage assay, with purified 3C^pro^ and peptide substrates, previously showed that the 3B3-3C boundary sequence is the most readily cleaved sequence *in trans* [38, 71]. The same study also found that replacing the P2 lysine residue with threonine prevented cleavage of a peptide substrate *in trans*. In our experiments, the reversion of the P2 residue from lysine (as found in 3B3/2) to threonine (as found in the wild-type P3 and 3B3/2 K>T), restored normal replication and processing. This observation is consistent with a switch from *cis* to *trans*-mediated processing. Thus the presence of fully processed 3C^pro^ or 3C^pro^-containing precursors may facilitate a *trans* mediated P3 cleavage pathway allowing temporal regulation of the appearance of alternative polyprotein precursors. Our current research goal is to fully understand the complexities of polyprotein processing and its importance for replication.

## Materials and methods

### Cell lines

BHK-21 cells obtained from the ATCC (LGC Standard) were maintained in Dulbecco’s modified Eagle’s medium with glutamine (Sigma-Aldrich) supplemented with 10% FCS, 50 U / ml penicillin and 50 μg / ml streptomycin.

### Plasmid constructs

The FMDV replicon plasmids, pRep-mCherry and pRep-ptGFP, along with equivalent constructs containing the 3D^pol^-GNN replication-defective point mutation to the 3D^pol^ polymerase have already been described [57].

Removal of individual copies of 3B was performed by two-step overlapping PCR mutagenesis using pRep-mCherry as a template with matched forward and reverse 3B mutagenic primers with flanking PCR primers FMDV_4728_45 and FMDV_7200_7180. The second round PCR product was generated using the products of the first round PCR reactions in combination with flanking PCR primers. The second round PCR product was digested with *Sac*I and used to replace the equivalent 3B containing *Sac*I fragment from pRep-mCherry. A similar strategy was used to introduce Y3F point mutations into individual copies of 3B and mutate the C-terminal residues of 3B2 and 3B3. To exchange the position of 3B2 and 3B3 in the replicon, a synthetic DNA fragment was purchased (IDT) representing the *Sac*I–*Sac*I fragment from pRep-mCherry with the sequence of 3B2 and 3B3 exchanged and used to replace the equivalent fragment from pRep-mCherry. Removing the IRES was performed by one-step PCR with primer FMDV_DeltraIRES_Xbal_fwd which was digested with *Xba*I before ligation into *Xba*I digested pRep-mCherry plasmids.

The pcDNA3 based expression plasmids used for coupled *in vitro* transcription and translation assays expressed FMDV P3 and mutants thereof were constructed by PCR. Briefly, the appropriate P3 DNA sequence was amplified from the relevant pRep-mCherry replicon using primers FMDV_3A_ATG_fwd or FMDV_3B_ATG_fwd or FMDV_3C_ATG_fwd (as appropriate) with FMDV_3D_NotI_rvs to include a Kozak modified translational start site and flanking unique *Not*I restriction sites. The PCR product was digested with *Not*I and cloned into *Not*I digest pcDNA3.1(+) (Thermo Fisher Scientific). The sequences of all plasmids were verified by DNA sequencing. The sequence of the primers used in this study are available on request.

### *In vitro* transcription

Replicon plasmid DNAs linearized with *Asc*I (NEB) were used in T7 *in vitro* transcription reactions as previously descried [60]. *In vitro* transcription reactions were incubated at 32°C for 4 hours, followed by treating with RQ1 DNase (Promega) and recovery of RNA using an RNA Clean and Concentrator– 25 spin column (Zymo Research), following manufacturer’s instructions. Transcripts were quantified and integrity assessed by denaturing MOPS/formaldehyde gel electrophoresis prior to transfection.

### Replication and complementation assays

BHK-21 cells seeded into 24-well tissue cultures plates were allowed to adhere for 16 hours before transfection in duplicate with *in vitro* transcribed replicon transcripts using Lipofectin reagent as previously described [60]. Fluorescent reporter protein expression was monitored using an IncuCyte Zoom Dual Colour FLR (Essen BioSciences), within a 37°C humidified incubator, scanning hourly up to 24 hours post transfection, unless otherwise stated. Images were captured and analysed using the associated software for fluorescent protein expression, as previously described [60]. Control transfections (un-transfected and the 3D-GNN transfection for input translation) were used to determine fluorescent thresholds and identify positive objects from background fluorescence. A positive object was determined as having an average fluorescent intensity of >8 green calibration units (GCU; an arbitrary fluorescent unit) and >2 RCU (red calibration units), which were kept constant throughout the experiments. The number of positive cells per well was determined from the average of up to nine non-overlapping images per well. Unless stated otherwise, data are presented as mean fluorescent positive cells per well at 8 hours post transfection when replication was approximately maximal. For each experiment the data were analysed as both fluorescent cell counts per well and total fluorescent intensity per well. There was no difference observed when the data were analysed in either way. Unless otherwise stated, statistical analysis was performed using a two-tailed unpaired t test.

### Passage of FMDV replicons

BHK-21 cells co-transfected with *in vitro* transcribed replicon transcripts were harvested at 8 hours post transfection and polyadenylated RNA extracted using a Dynabeads mRNA DIRECT purification kit (Thermo Fisher Scientific) following manufacturer’s instructions.

Naïve BHK-21 cells seeded into 24-well tissue cultures plates were allowed to adhere for 16 hours before duplicated wells were transfected with 100 ng of the purified ‘passage 1’ polyadenylated RNA using Lipofectin reagent, following the manufacturer’s instructions. Fluorescent reporter protein expression was monitored using an IncuCyte Zoom Dual Colour FLR (Essen BioSciences) and analysed using the associated software for fluorescent protein expression as described above.

### Construction of recombinant viruses

All replicons used in this study are based on plasmid pT7S3 [72] which encodes a full-length infectious copy of FMDV O1 Kaufbeuren. The coding region for the reporter and flanking FMDV sequence were excised from selected replicons by DNA digestion with *Psi*I and *Xma*I restriction sites and replaced with the corresponding fragment from pT7S3 encoding the capsid region. The resulting infectious copy plasmids (ICP) were verified by restriction digest analysis. Infectious copy plasmids were linearized by digestion with *Asc*I and full-length viral RNA transcribed using a T7 MEGAscript kit (Thermo Fisher Scientific) and subjected to DNase digestion using TurboDNase (Thermo Fisher Scientific) followed by purification using a MEGAclear Transcription Clean-Up kit (Thermo Fisher Scientific). RNA quality and concentration were determined by denaturing agarose gel electrophoresis and nanodrop.

BHK-21 cells were transfected in a 25 cm^2^ flask with 8 μg ICP-derived RNA using TransIT transfection reagent (Mirus) according to the manufactures protocol. At 24 hours post-transfection, cell lysates were prepared by freeze/thawing overnight and the lysate clarified by centrifugation. The clarified lysate (1 ml) was passaged blind onto subsequent BHK-21 cell monolayers (~70% confluent) in 25 cm^2^ flasks in 5 ml of virus growth medium (reduced serum medium with 1% FCS) for a maximum of 48 hours. Flasks were scored for the appearance of cytopathic effect (CPE) indicating recovery of infectious virus. The cells were freeze/thawed and the resulting lysate clarified by centrifugation and stored at -80°C.

### Sequencing of recovered viruses

Recovered viruses (at BHK-21 p4), were sequenced on the Illumina Miseq (Illumina) platform using a modified version of a previously described PCR-free protocol [73]. Briefly, total RNA was extracted from clarified infected cell lysates using TRIzol Reagent (Thermo Fisher Scientific) as per manufacturer’s instructions. Any residual genomic DNA was removed using DNA-free DNA Removal Kit (Thermo Fisher Scientific) following manufacturer’s protocol. After precipitation with 3 M sodium acetate and ethanol, 10 μl (containing from 1 pg to 5 μg) of RNA was used in a reverse transcription (RT) reaction as previously described [74] with the exception that, in addition to Random Hexamers (Bioline Reagents Ltd), two primers (Rev6 and NK72, previously described [73]) were included in the first incubation step, and the final incubation step at 42°C was carried out for 40 minutes. Second strand synthesis was carried out using the NEBNext mRNA Second Strand Synthesis Module (NEB) following the manufacturer’s protocol and subsequent cDNA extracted by the addition of an equal volume of phenol:chloroform:isoamyl alcohol (Thermo Fisher Scientific) followed by 3 M sodium acetate/ethanol precipitation as described [74]. cDNA was quantified using the Qubit dsDNA HS Assay Kit (Thermo Fisher Scientific) as per manufacturer’s instructions and a cDNA library was prepared using the Nextera XT DNA Sample Preparation Kit (Illumina) following manufacturer’s recommendations. Sequencing was carried out on the MiSeq platform using Miseq Reagent Kit v2 (300 cycles) chemistry (Illumina).

The quality of subsequent FastQ files was checked using FastQC (available at http://www.bioinformatics.babraham.ac.uk/projects/fastqc/) and poor quality reads were filtered out using the Sickle algorithm ([75]; available at https://github.com/najoshi/sickle). Host cell reads were removed using the FastQ Screen algorithm (available at http://www.bioinformatics.babraham.ac.uk/projects/fastq_screen/) and FMDV reads assembled de novo into contigs with IDBA-UD [76]. Only contigs which matched the FMDV library after running a Basic Local AlignmentSearch Tool (BLAST) algorithm [77] were assembled into consensus sequences using SeqMan Pro software implemented in the DNA STAR Lasergene 13 package (DNASTAR). To confirm the consensus, reads were mapped using Burrows-Wheeler Aligner (BWA) [78]. Mapped reads were visualised using Integrative Genomics Viewer (IGV) [79].

### Immunoblotting

Immunoblotting was performed as previously described [80]. The primary antibodies rabbit anti-3D^pol^ 397 polyclonal, mouse anti-3B 1F8 monoclonal, mouse anti-3C 2D2 and mouse anti-3A 2C2 monoclonal (all generous gift from Professor Sobrino, Madrid) have been previously described [60]. The rabbit anti-3B1 -3B2 and -3B3 specific antibodies were generated for this study on our behalf by 21^st^ Century Biochemicals (Twentyfirst Century Biochemicals, Inc). Briefly, rabbits were immunised with VPg specific peptides (VPg1: C-Ahx-VRAKLPQQE-OH, VPg2: Ahx-VKAKAPVVKE-OH, Ahx-VPg3: VKAKNLIVTW-OH, all generated by 21st Century Biochemicals), and serum affinity purified by immunodepletion against the other VPg peptides. Cross-reactivity was checked by dot blot and immunodepletion repeated until the final antibody showed no VPg cross reactivity. The specificity of each VPg-specific antibody was confirmed by ELISA against the peptides used for immunisation.

### Coupled *in vitro* transcription and translation assay

Coupled *in vitro* transcription and translation assays were performed using the TNT Quick Coupled Transcription/Translation System (Promega) following manufacturer’s protocols. Briefly, 12.5 μl reactions were assembled on ice containing 500 ng of pcDNA T7 expression plasmid and 0.4 mCi / ml of [^35^S] methionine (PerkinElmer). Reactions were incubated at 30°C for 40 minutes before addition of 1 μl of 50 mg / ml unlabelled methionine and cysteine. At 20 minute intervals samples of each reaction were stopped by the addition of 2 x Laemmlli buffer. The radiolabelled products were separated by SDS-PAGE before visualisation of cleaved proteins by autoradiography.

## Supporting information

S1 Fig Residues at the 3B-3C boundary are important for replicon replication. BHK-21 cells seeded into 24 well plates were transfected with mCherry replicons bearing 3B deletions or mutations as well as wild-type (wt) and replication-defective polymerase mutant (3D^pol^-GNN) controls. Transfections were performed with replicons from which 3B1, 3B2 or 3B3 had been deleted (Δ3B1, Δ3B2, and Δ3B3, respectively), both 3B1 and 3B2 deleted in tandem (Δ3B1+2) or point-mutation to the uridylatable tyrosine of 3B1, 3B2 or 3B3 (3B1^Y3F^, 3B2^Y3F^ or 3B3^Y3F^, respectively). Expression of mCherry was monitored hourly over a 24 hour period. Data shown represent mean mCherry positive cells per well at hourly timepoints on (A) a linear scale and (B) a log_10_ scale. For clarity, the error bars have been removed (n = 3).
(TIF)

S2 Fig Mutations at the 3B-3C boundary disrupt P3 polyprotein processing. Plasmid constructs expressing wild-type FMDV P3 or the 3B3/2 mutant polyprotein were used to assemble coupled transcription/translation reactions with [^35^S] labelled methionine. At 20 minute intervals samples were taken and the reaction stopped by the addition of 2 x Laemmli buffer. Protein products were separated on 12% SDS-PAGE and probed by Western blot for expression of FMDV non-structural protein expression.
(TIF)

S3 Fig Mutations at the C-terminus of 3B3 prevent complementation of 3D^pol^ mutations but not 3B mutations *in trans*. **(A)** BHK-21 cells seeded into 24-well plates were co-transfected with mCherry replicons bearing replication-defective 3B or 3D^pol^ mutations or controls and wild-type ptGFP, ptGFP-3B123^Y3F^ or ptGFP-3D^pol^-GNN replicon. In **(A)** all the mCherry replicons contained a full IRES. In **(B)** the mCherry replicons contained a deletion to the entire IRES (ΔIRES), in addition to the indicated non-structural protein mutation (3D^pol^-GNN, 3B123^Y3F,^ 3B3/2). Expression of mCherry is shown representing mean positive cells per well at 8 hours post transfection (n = 3, ± SD).
(TIF)
