# Supplementary figures and images for: Genetic economy in picornaviruses: Foot-and-mouth disease virus replication exploits alternative precursor cleavage pathways
Source: PLoS Pathog. 2017 Oct 2;13(10):e1006666. doi: 10.1371/journal.ppat.1006666 (PMC5638621; doi:10.1371/journal.ppat.1006666)

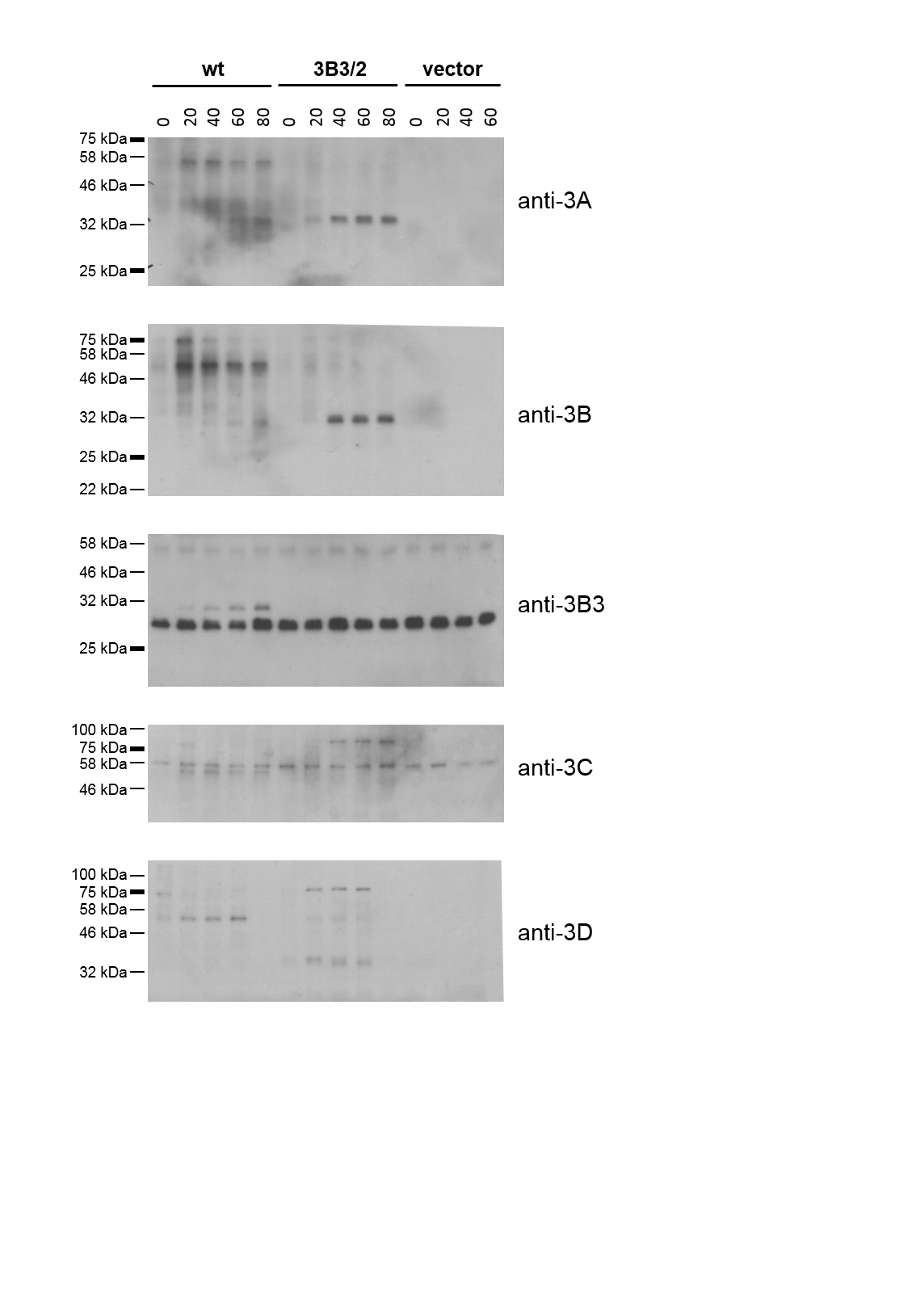

Supplement: S2 Fig — Plasmid constructs expressing wild-type FMDV P3 or the 3B3/2 mutant polyprotein were used to assemble coupled transcription/translation reactions with [35S] labelled methionine. At 20 minute intervals samples were taken and the reaction stopped by the addition of 2 x Laemmli buffer. Protein products were separated on 12% SDS-PAGE and probed by Western blot for expression of FMDV non-structural protein expression. (TIF) [file ppat.1006666.s002.tif]

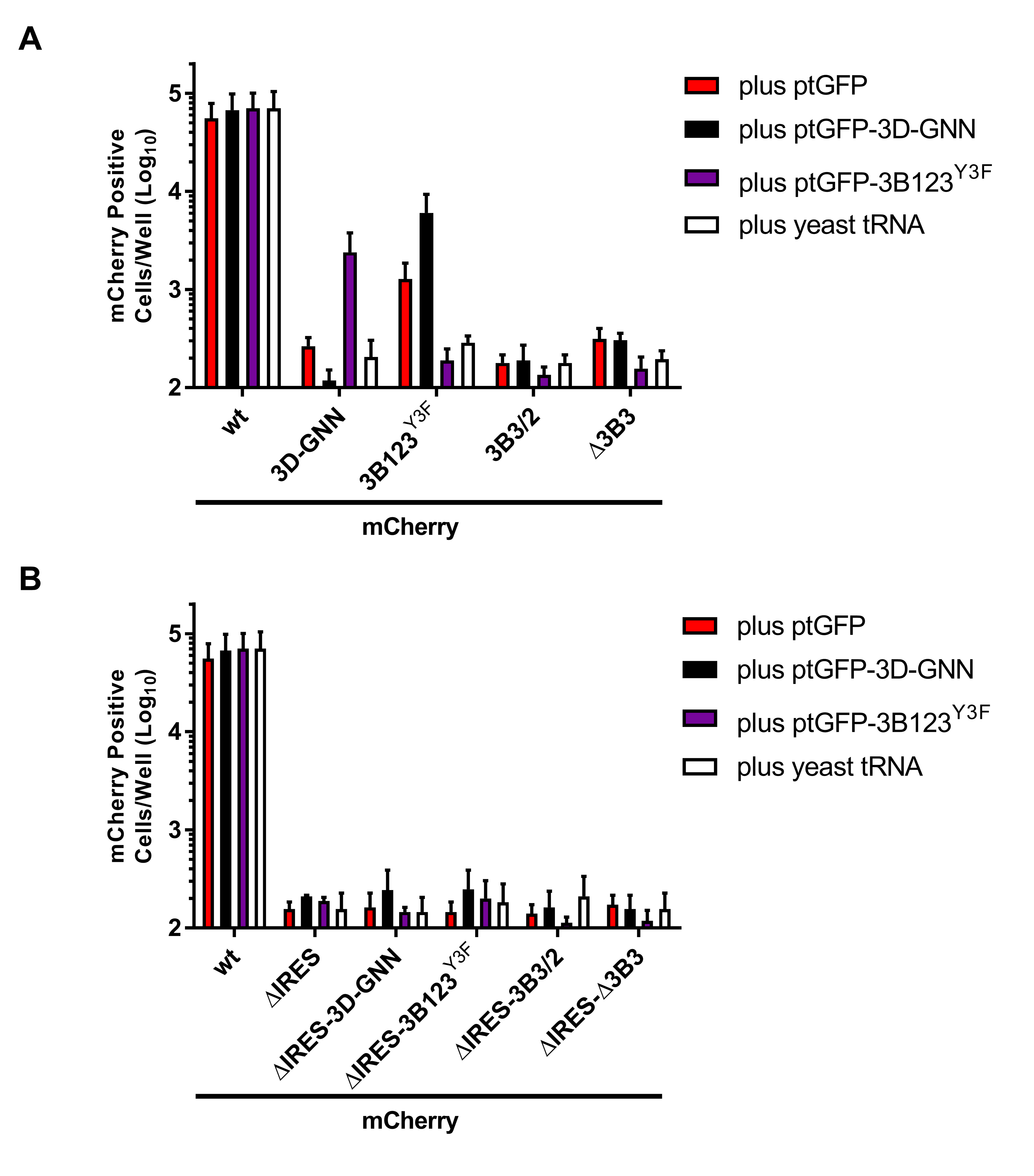

Supplement: S3 Fig — (A) BHK-21 cells seeded into 24-well plates were co-transfected with mCherry replicons bearing replication-defective 3B or 3Dpol mutations or controls and wild-type ptGFP, ptGFP-3B123Y3F or ptGFP-3Dpol-GNN replicon. In (A) all the mCherry replicons contained a full IRES. In (B) the mCherry replicons contained a deletion to the entire IRES (ΔIRES), in addition to the indicated non-structural protein mutation (3Dpol-GNN, 3B123Y3F, 3B3/2). Expression of mCherry is shown representing mean positive cells per well at 8 hours post transfection (n = 3, ± SD). (TIF) [file ppat.1006666.s003.tif]
